# Improving the Detection of Hepatocellular Carcinoma Using Serum AFP Expression in Combination with GPC3 and Micro-RNA MiR-122 Expression

**DOI:** 10.1515/biol-2019-0007

**Published:** 2019-03-20

**Authors:** Jian Li, Sun Qiyu, Tiezheng Wang, Boxun Jin, Ning Li

**Affiliations:** 1 Department of Hepatobiliary Surgery, YouAn Hospital Affiliated to Capital Medical University, 8 Xitoutiao Road, Fengtai District, Beijing, 100069, China; 2 Department of Hepatobiliary Surgery, Hospital Affiliated to Chengde Medical University, 36 Nanyingzi Road, Chengde, 067000, China

**Keywords:** Hepatocellular carcinoma, Alpha-fetoprotein, Glypican-3, miR-122, Hepatitis C virus, Liver fibrosis

## Abstract

Early diagnosis of hepatocellular carcinoma (HCC) greatly improves the survival and prognosisfor patients. In this study weevaluate the diagnostic promise of combining serum alpha-fetoprotein (AFP) expression with two potential biomarkers, serum glypican-3 (GPC3) and expression of the micro-RNA miR-122 for hepatitis C virus (HCV) related early-stage HCC. For this study serum samples from 47 patients with early-stage HCC, 54 chronic HCV (CH) carriers, 35 patients with liver cirrhosis (LC) and 54 health controls (HC) were collected. In addition to routine laboratory investigations, serum AFP, GPC3 and miR-122 were measured in all patients and healthy controls. Receiver operating characteristic (ROC) curves were used to present sensitivity and specificity for the biomarkers. The three markers were all significantly elevated in the serum samples from HCC patients. ROC curves showed the three markers had similar diagnostic capacities for distinguishing early-stage HCC from HCV-positive controls (LC + CH). In order to distinguish early-stage HCC from high-risk LC patients, the expression of miR-122 was superior to GPC3. Combination of the three markers as a panel showed a better diagnostic performance than any of the single markers (P <0.05). Overall, this study revealed that serum expression of GPC3 and miR-122 may be useful biomarkers to combine with serum AFP expression for the diagnosis of HCV related early-stage HCC.

## 1 Introduction

Hepatocellular carcinoma (HCC) is the most prevalent type of liver cancer [1]. In addition, HCC frequently exhibits a very poor prognosis in patients [1]. In China, HCV infection has been recognized as a central cause of chronic liver disease, cirrhosis, and HCC secondary to hepatitis B virus [2, 3, 4]. In the past 10 years the incidence of HCV infection has increased from 0.7 to 15.0 cases per 100 000 persons [3].

While great progress has been made in HCC treatment, the prognosis for HCC remains poor and adverse effects of chemotherapy are common [1]. Early diagnosis of HCC and timely treatment can greatly improve life expectancy and reduce mortality. Currently, serum AFP expression is the most well established serum biomarker for the diagnosis of HCC. However, the diagnostic performance of AFP expression in detecting early-stage HCC is suboptimal [5, 6]. Therefore, the identification of additional effective and reliable non-invasive biomarkers for the diagnosis of early-stage HCC is of important clinical significance.

Glypican-3 (GPC3) is widely expressed in human embryos and involved in human tissue growth. GPC3 can be detected in the fetal liver, but cannot be identified in any normal adult hepatic tissue [7, 8]. In recent years, GPC-3 expression levels have been found to be elevated in HCC patients, as shown by immunohistochemistry [9]. The micro-RNA miR-122 plays a role in regulating lipid and cholesterol metabolism, and comprises 70% of the total miRNAs in the liver. Furthermore, miR-122 is important for HCV stability in liver cells and is known to regulate HCV stability by binding to an unstructured 5’ UTR of HCV-RNA [10]. It has also been found that miR-122 is involved in regulating multiple target genes associated with HCC pathogenesis [11].

The combination of multiple biomarkers can greatly improve the ability of both the detection and diagnosis of disease and previous studies have reported that a combination of biomarkers is superior in detection of hepatocellular carcinoma [12, 13]. The diagnostic capacity of combination of AFP with GPC3 and miR-122 for the detection and diagnosis of HCC is unclear. In the present study, we investigated and evaluated the diagnostic capacity of serum GPC3 and miR-122 expression in combination with serum AFP expression for HCV-related early-stage HCC.

## 2 Materials and Methods

### 2.1 Subjects

HCV carriers and liver cirrhosis (LC) patients were recruited from our institution during the study period. These patients had been positive for HCV-RNA in sera for more than 6 months. Informed consent has been obtained from all individuals included in this study. The diagnosis of HCC was based on American Association for the Study of Liver Diseases (AASLD) Practice Guidelines [14] and staged by the Barcelona Clinic Liver Cancer (BCLC) system [15]. BCLC stage 0 + A HCC was defined as early-stage HCC and 47 patients with early-stage HCC were included in our study. Patients with chronic HCV infection or cirrhosis were designated as controls. LC was diagnosed by histological examination of liver biopsy and imaging findings. In addition, 54 healthy volunteers without liver diseases and any tumors were recruited as healthy controls.

**Ethical approval**: The research related to human use has been complied with all the relevant national regulations, institutional policies and in accordance the tenets of the Helsinki Declaration, and has been approved by the ethics committee of YouAn Hospital Affiliated to Capital Medical University.

**Informed consent**: Informed consent has been obtained from all individuals included in this study

### 2.2 Laboratory tests

Before any curative treatment, patient blood samples were obtained. After centrifugation at 3000 rpm for 15 min at room temperature, the supernatant was transferred into a new tube. The supernatant was then centrifuged at 15,000 rpm using a high speed refrigerated centrifuge for 30 min to precipitate cell debris and then stored at -80°C. Serum AFP levels were measured using a chemiluminescent immunoassay (ELICA) [16]. Serum GPC3 levels were measured using an ELISA method [17] (Shanghai LangDun Biological Technology Co., LTD) according to the manufacturer’s instruction.

### 2.3 Reverse transcription and qPCR

The total RNA was extracted by using TRIzol reagent (Invitrogen) according to the manufacturer’s instructions. Synthetic miRNA cel-miR-39 (Rubo, China) served as an internal control was added to each denatured sample at a final concentration of 10^-4^ pmol/L. The RNA concentration was quantified with a DU 800 spectrophotometer (Beckman Coulter). Reverse transcription was carried out using a TaqMan microRNA Reverse Transcription Kit for the following reaction: 1.5 μL of 10×Reverse Transcription Buffer, 4.16 μL of nuclease-free water, 0.19 μL of RNaseinhibitor, 0.15 μL of 100 mmol/L dNTPs, 1 μL of Multiscribe Reverse Transcriptase, 5 μL of total RNA and 3 μL of stem-loop RT primer. The RT reaction was performed at 16°C for 30min, 42°C for 30min and 85°C for 5 min by using the Applied Biosystems 7300 Sequence Detection System. qPCR was subsequently carried out using a TaqMan MicroRNA assay (Applied Biosystems) for the following reaction: 10 μL of 2×Taq Manuniversal PCR Master Mix II, 1 μL of 20×TaqMan small RNA Assay reagent, 7.67 μL of nuclease-free water and 1.33 μL of product from the RT reaction. The qPCR was performed at 95°C for 10 min, 40 cycles of 95°C for 15 s, and 60°C for 1 min. Fluorescence readings were taken during the 60°C step. A no template control (NTC) and a control without reverse transcription (NRT) were used as negative controls. To calculate the relative expression levels of miR-122, cel-miR-39 was used as a control miRNA and the 2^-ΔCt^ (ΔCt =Ct miR-122− Ct cel-miR-39) method was employed to assess miRNA expression [18, 19].

### 2.4 Statistical analysis

miR-122 values were normalized to cel-miR-39 and are expressed as 2^-ΔCT^ [19]. The quantitative data are presented as means ± standard deviations. All continuous variables were checked using the Kolmogorov-Smirnov normality test to show their distributions. Continuous variables with normal distributions were compared using unpaired Student’s t-tests and analysis of variance (ANOVA). Continuous variables with abnormal distributions were compared using Mann-Whitney U tests and ANOVA. For categorical variables, the chi-square test was used. Receiver operating characteristic curves were used to present sensitivity and specificity for the biomarkers. The correlation between marker concentrations was analyzed with Pearson’s χ^2^ test. To assess the diagnostic performance of the combination of biomarkers, logistic regression models including two or three markers as covariates were performed [13]. P values <0.05 were considered statistically significant. Statistical calculations were performed using SPSS 16.0 and medcale software.

## 3 Results

According to BCLC stage, 47 patients with early-stage HCC (10 patients with stage 0 HCC and 37 patients with stage A HCC) among 47 HCC patients were collected. We categorized the subjects into 4 groups: a healthy control group (HC, n=54), a chronic HCV carrier group (CH, n=54), a LC group (LC, n=35), and an early-stage HCC (n=47) group. (Table 1)

**Table 1:** Demographic and clinical data of HCC and non-malignant chronic liver disease patients

Parameter	HC (n=54)	CH (n=54)	LC(n=35)	HCC (n=47)
Age (years)	45±15	37±12	55±8	63±10
Male, n(%)	23(42.59)	31(57.41)	22(62.86)	31(65.96)
ALT (U/l)	26±20	39±27	84±88	79±42
AST (U/I)	26±18	35±13	69±44	65±36
Total bilirubin (mg/dl)	7±8	14±9	28±23	24±11
Albumin (g/dl)	43±4	43±4	38±5	36±5
Hemoglobin (g/dl)	146±17	143±19	123±36	126±24
Platelet count (×10^9^/l)	212±42	196±45	107±45	108±55
BCLC stage, n				
(%)				
Stage 0				20 (43)
Stage A				27 (57)

ALT: alanine aminotransferase; AST: aspartate aminotransferase

### 3.1 Expression of serum GPC3, miR-122 and AFP in patients

**Fig.1:**
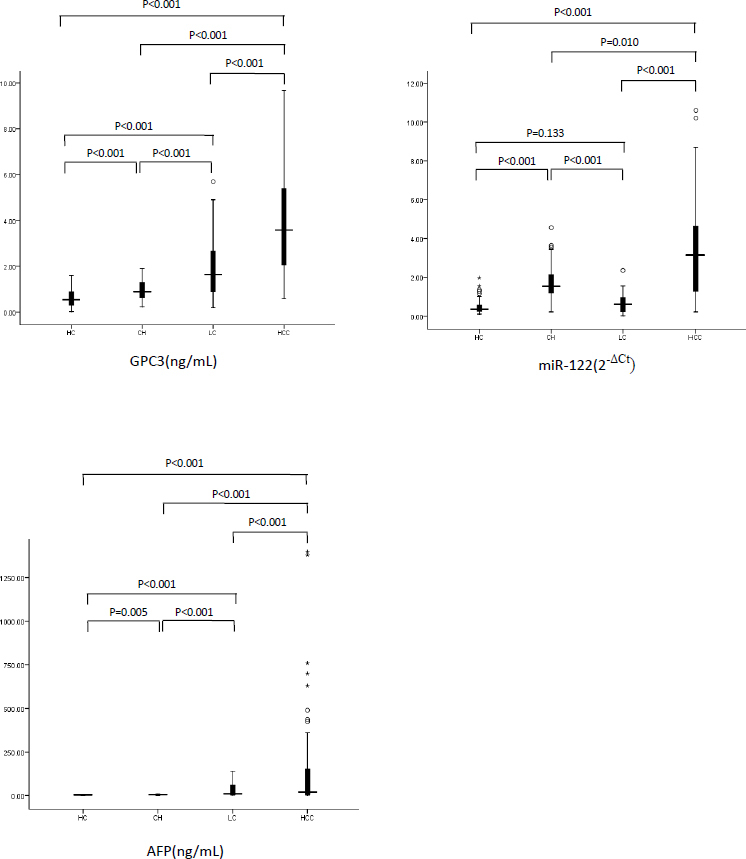
Serum concentration of GPC3, miR-122 and AFP in HC, CH, LC and HCC groups. (HC health control, CH chronic hepatitis C carriers, LC liver cirrhosis, HCC hepatocellular carcinoma)

We investigated the levels of expression of serum AFP, GPC3, and miR-122 levels in patients with CH, LC, and HCC (Table 2; Fig.1). GPC3 levels were elevated significantly in HCC patients compared to healthy controls (p<0.001), CH patients (p <0.001) or LC patients (p<0.001). MiR-122 and AFP levels were also significantly upregulated in HCC patients and p values were <0.001 compared to subjects in other groups including healthy controls, CH patients and LC patients.

**Table 2:** Serum AFP, GPC3 and miR-122 levels in different groups

Parameter	HC (n=54)	CH (n=54)	LC(n=35)	HCC (n=47)	P value
AFP (ng/mL)	3.7±1.1	4.5±1.8	33.3±40.61	171.7±324.7	<0.001
GPC3 (ng/mL)	0.6±0.4	1.0±0.4	2.0±1.4	3.9±2.4	<0.001
miR-122 (2^-ΔCt^)	0.5±0.4	1.6±0.9	0.7±0.5	3.5±2.6	<0.001

### 3.2 Optimum cutoff values of serum GPC3, miR-122 and AFP

**Fig.2:**
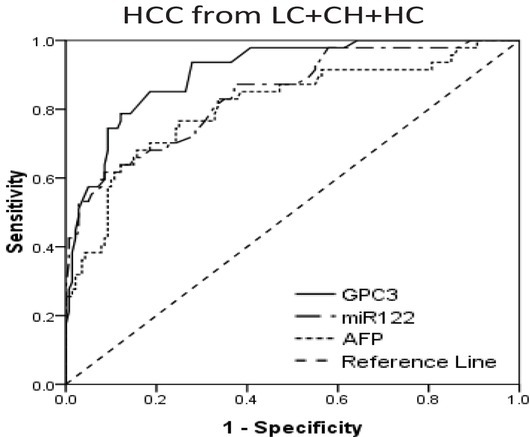
ROC curve for AFP, GPC3 and miR-122 for discriminate early-stage HCC from all controls (LC+CH+HC). AUC for GPC3, miR-122 and AFP were 0.909, 0.844 and 0.810 respectively. The optimum cutoff values of GPC3, miR-122 and AFP were 1.75 ng/ml, 2.22 and 7.33 ng/ml respectively. (ROC, receiver operating characteristic, AUC area under ROC curve, LC liver cirrhosis, CH chronic hepatitis C carriers, HC health control, HCC hepatocellular carcinoma)

ROC curves were performed to determine the diagnostic values and optimum cutoff of GPC3, miR-122 and AFP for early-stage HCC from all controls (LC+CH+HC, patients of liver cirrhosis and chronic hepatitis C carriers and health controls). The AUC of GPC3 was 0.909 (95% CI 0.864-0.954) and the optimum cutoff of GPC3 was 1.75 ng/ml with a sensitivity of 78.72%, and a specificity of 87.86%. The AUC of miR-122 was 0.844 (95% CI 0.778-0.911) and the optimum cutoff of miR-122 was 2.22 with a sensitivity of 62.70%, and a specificity of 91.43%. The AUC of AFP was 0.810 (95% CI 0.732-0.888) and the optimum cutoff of AFP was 7.33 ng/ml with a sensitivity of 68.09%, and a specificity of 84.29%. When the cutoff of AFP was 7.33 ng/ml, 16 patients were AFP-negative among 47 early-stage HCC patients (Fig.2).

### 3.3 Combination of the three markers for diagnosis of early-stage HCC

**Fig.3:**
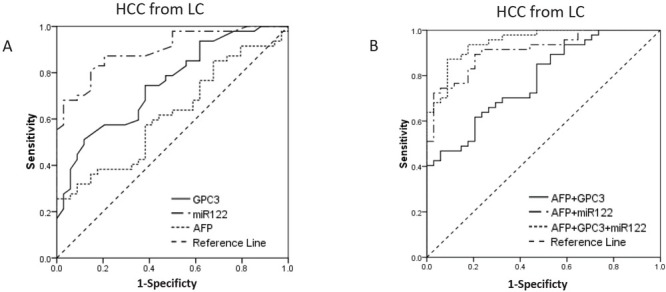
ROC curve for AFP, GPC3 and miR-122, and their combinations for discriminate early-stage HCC from HCV positive controls (LC+CH). A AUC for GPC3, miR-122 and AFP were 0.850, 0785 and 0.754 respectively. B AUC for combinations of AFP+GPC3, AFP+miR-122 and AFP+GPC3+miR-122 were 0.898, 0.849 and 0.947 respectively. Combination of the three biomarkers could improve AUC than AFP alone (P<0.05). (ROC, receiver operating characteristic, AUC area under ROC curve, LC liver cirrhosis, CH chronic hepatitis C carriers, HCC hepatocellular carcinoma)

The areas under the curves of GPC3, miR-122 and AFP for diagnosis of early-stage HCC from HCV-positive controls (LC+CH) were 0.850 (95% CI 0.781-0.920), 0.785 (95% CI 0.699-0.871) and 0.754 (95% CI 0.665-0.844) respectively. The three markers showed no significant different of AUC (Table 3; Fig.3). A binary logistic regression model was used to evaluate the diagnostic capacity of combined markers [13]. The AUC of combination of AFP and GPC3 was 0.898 (95% CI 0.847-0.949), larger than AUC of AFP or GPC3 alone (p = 0.010 and p = 0.038, respectively). Similarly, the AUC of combination of AFP and miR-122 was 0.849 (95% CI 0.769-0.923), larger than AUC of AFP or miR-122 alone (p = 0.048 and p = 0.045, respectively). When the three markers were combined, the diagnostic performance was best with AUC of 0.947 (95% CI 0.913-0.981) (Table 3; Fig.3).

**Table 3:** Diagnostic capacities of AFP, GPC3 and miR-122 for early-stage HCC

	Early-stage HCC from LC+CH				Early-stage HCC from LC			
	AUC	95%CI	Sensitivity(%) Specificity(%)		AUC	95%CI	Sensitivity(%)	Specificity(%)
GPC3	0.850	0.781-0.920	78.70	79.31	0.752	0.647-0.856	51.06	88.24
miR-122	0.785	0.699-0.871	61.68	86.23	0.900	0.835-0.964	97.23	79.41
AFP	0.754	0.665-0.844	68.11	74.2	0.618	0.496-0.739	36.17	85.29
AFP+GPC3	0.898	0.847-0.949	85.01	81.62	0.782	0.685-0.880	61.70	79.41
AFP+miR-122	0.849	0.769-0.923	73.34	90.83	0.911	0.850-0.972	72.34	95.06
AFP+GPC3+miR-122	0.947	0.913-0.981	89.43	85.12	0.949	0.907-0.992	87.234	91.176

**Fig.4:**
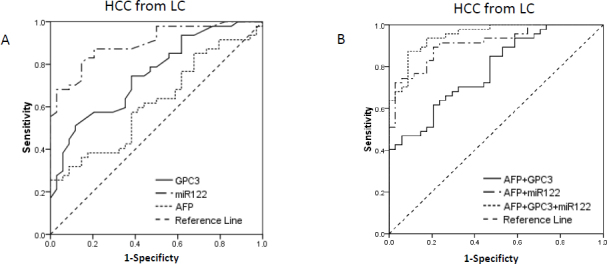
ROC curve for AFP, GPC3 and miR-122, and their combinations for discriminate early-stage HCC from LC. AUC for GPC3, miR-122 and AFP were 0.752, 0.900 and 0.618 respectively. AUC for combinations of AFP+GPC3, AFP+miR-122 and AFP+GPC3+miR-122 were 0.782, 0.911 and 0.949 respectively. Combination of the three biomarkers could improve AUC than AFP alone (P<0.05). (ROC, receiver operating characteristic, AUC area under ROC curve, LC liver cirrhosis, HCC hepatocellular carcinoma)

It is important to clinically distinguish between HCC and high-risk LC. Diagnostic capacities of serum GPC3, miR-122 and AFP were assessed to discriminate between HCC patients and LC patients. The AUC of miR-122 was largest of 0.900 (95% CI 0.835-0.964). Compared to GPC3 and AFP, miR-122 had better diagnostic capacity in discriminating early-stage HCC from high-risk patients (p=0.017 and p<0.001, respectively). The AUC of AFP was 0.618 (95% CI 0.496-0.739) in discriminating early-stage HCC from LC patients. The AUC of GPC3 was similar to that of AFP with AUC of 0.752 (95% CI 0.647-0.856) (p=0.091). Combination miR-122 with AFP could significantly enhance the diagnostic capacity with AUC of 0.911 (95% CI 0.850-0.972) than AFP itself (p<0.001), but not miR-122 (p=0.352). Similarly, the AUC of combination GPC3 with AFP was 0.782, lager than that of AFP itself (p=0.008), but not GPC3 (p=0.352). Combination of the three markers had higher AUC of 0.949 (95% CI 0.877-0.986) compared to combination of miR-122 and AFP (p=0.047) or combination of GPC3 and AFP (p<0.001). Combination of the three markers also had larger AUC than the single marker of miR-122 in discriminating early-stage HCC from LC patients (p=0.025) (Table 3; Fig.4).

In our study, we chose 7.33 ng/ml as the cutoff value of AFP, 1.75 ng/ml as the cutoff value of GPC3 and 2.22 as the cutoff value of miR122. When the cutoff of AFP was 7.33 ng/ml, 16 patients were AFP-negative among 47 early-stage HCC patients. 12(75%) of 16 AFP-negative HCC patients had positive GPC3 (>1.75 ng/ml), and 9 (56%) of them had positive miR122 (>2.22). 14 (88%) patients had either positive GPC3 or positive miR122 results. (Table 4)

**Table 4:** Sensitivity of GPC3 and miR-122 in AFP-negative early-stage HCC patients

n	GPC3(%)	miR-122(%)	Combination of GPC3 and miR-122 (%)
16	12(75)	9(56)	14(88)

### 3.4 Correlation analysis

No significant correlations were found between any 2 of the 3 markers. In the CH group, levels of miR-122 were significantly correlated with aspartate transaminase (AST, p = 0.025) and alanine transaminase (ALT, p = 0.027), which are known as liver necro-inflammatory markers.

## 4 Discussion

The diagnosis of HCC at an early stage is essential for improving patient survival. Imaging examinations and biopsies are the major means for diagnosis of HCC at present. But imaging examinations cannot easily distinguish between benign tumors and malignant cancer [20] and biopsies have the risk of tumor cells seeding along the needle track [21]. So biomarkers in peripheral blood which can efficiently distinguish HCC are urgently needed.

AFP is the most widely used tumor marker, but its sensitivity and specificity is not sufficient [5, 6]. Elevated GPC3 expression in primary carcinoma of the liver was first reported by Hsu et al. [22]. Since then, the role of GPC3 in diagnosis, progression, and treatment of HCC *in vivo* and *in vitro* has been studied extensively. Studies have shown that GPC3 could increase the c-Myc expression and GPC3 was involved in the occurrence and development of HCC. [23, 24]. Although GPC3 as a specific marker of liver cancer has been studied widely, the diagnostic capacity of serological GPC3 for HCC is controversial [25 , 26, 27]. In the present study, we evaluated GPC3 expression patterns during HCV-related disease progression. We compared GPC3 levels between different groups. Our data showed the level of GPC3 was higher in CH patients compared to controls, and increased significantly in LC patients compared to CH patients. In HCC patients the level of GPC3 was highest. The expression patterns of GPC3 were similar to those of AFP and are consistent with the results of a tissue expression signature study [28]. Another biomarker is miR-122, which demonstrated elevated expression in CH patients compared to controls and miR-122 was significantly correlated with AST and ALT in CH patients, consistent with previous studies [4, 29]. The levels of miR-122 decreased in LC patients compared to CH patients, then elevated in HCC patients and the level of miR-122 in HCC was highest. Our results were consistent with previous studies which have reported higher levels of serum miR-122 in HCC patients than LC patients or healthy controls [30, 31, 32, 33]. But in HCC tumor tissues, miR-122 expression was reportedly downregulated [34]. It may be postulated that these contrary results showing low levels of miR-122 in tumor tissues was due to its increased release [33, 35], however it is clear that the exact mechanisms requires further study.

The diagnosis of HCC at early stage is very important for clinical treatment. So we chose patients with early-stage HCC defined as stages 0 + A according to BCLC staging system as study subjects. ROC curves showed AFP, GPC3 and miR-122 had similar diagnostic capacities for distinguishing early-stage HCC from HCV-positive controls (LC+CH). The combination of AFP with GPC3 or the combination of AFP with miR-122 could both increase the diagnostic capacity of AFP with larger AUC. The combination of all three markers had the largest AUC, with a sensitivity of 89.43% and specificity of 85.12%. In 16 AFP-negative (AFP<7.33ng/ml) HCC patients, 12 (75%) patients had higher levels of GPC3 and 9 (56%) patients had higher levels of miR-122. It showed in patients with normal levels of AFP, the examinations of GPC3 and miR-122 could increase the diagnostic sensitivity of early-stage HCC.

Patients with LC are known to be at high risk of developing HCC. Early diagnosis of HCC among these patients is important to reduce the mortality of the disease [36]. ROC curves showed AFP and GPC3 were not effective in distinguishing between HCC from LC patients with AUC of 0.618 and 0.752, respectively. The diagnostic sensitivities of the two markers were low with 36.17% for AFP and 51.06% for GPC3, respectively. The expression of miR-122 was a better biomarker in distinguishing HCC from LC patients with AUC of 0.900. It has been reported that miR-122 expression negatively correlates with liver fibrosis [29, 37]. With the development of fibrosis, more myelofibroblasts proliferate and more extracellular matrix accumulates. These factors cause the gradual loss of hepatocytes, followed by falling miR-122 levels [29, 37]. But in HCC patients, serum miR-122 increased significantly and the serum miR-122 had better diagnostic capacity than AFP and GPC3 in distinguishing early-stage HCC from high-risk LC patients. Our findings were in agreement with Zekri et al. [38] and Varnholt et al. [39] which both reported higher levels of serum miR-122 in HCV-related HCC than controls. Combination of the three markers could enhance the diagnostic capacity of HCC in these high-risk patients. The AUC of combination of AFP, GPC3 and miR-122 was larger than that of any single maker including miR-122.

Our current study confirmed that serum levels of GPC3 and miR-122 are useful markers for the diagnosis of HCV-related early-stage HCC. In patients with a background of cirrhosis due to chronic HCV infection, miR-122 was a superior biomarker. The combination of AFP, GPC3 and miR-122 as a panel for diagnosis of HCC could enhance the diagnostic sensitivity of a single marker, such as AFP alone. Patient numbers in our study were relatively small, and future studies should engage larger numbers of patients to confirm these observations. The biological actions of GPC3 and miR-122 in the pathogenesis of HCC and their prognostic values in HCC outcome also deserve further study.
